# Energy metabolism of the equine cumulus oocyte complex during *in vitro* maturation

**DOI:** 10.1038/s41598-020-60624-z

**Published:** 2020-02-26

**Authors:** N. Lewis, K. Hinrichs, H. J. Leese, C. McG. Argo, D. R. Brison, R. Sturmey

**Affiliations:** 1 Institute of Ageing and Chronic Disease, University of Liverpool, Cheshire, UK; 2 Texas A&M University, College Station, Texas USA; 3 Cardiovascular and Metabolic Research, Hull York Medical School, University of Hull, Hull, Yorkshire UK; 4 Maternal and Fetal Health Research, Faculty of Biology, Medicine & Health, University of Manchester, Manchester Academic Health Science Centre, Manchester, UK; 5 Present Address: Scotland’s Rural College (SRUC), Northern Faculty, Craibstone Campus, Aberdeen, AB21 9YA Scotland

**Keywords:** Developmental biology, Embryology, Metabolism

## Abstract

Horses are one of the few species, beside humans, in which assisted reproductive technology has important clinical applications. Furthermore, the horse can serve as a valuable model for the study of comparative reproductive biology. Here we present the first comprehensive characterisation of energy metabolism and mitochondrial efficiency in equine cumulus-oocyte complexes (COCs) during *in vitro* maturation (IVM), as determined using a combination of non-invasive consumption and release assays and mitochondrial function analysis. These data reveal notable species-specific differences in the rate and kinetics of glucose consumption and glycolysis throughout IVM. Approximately 95% of glucose consumed was accounted for by lactate production; however, high concurrent oxygen consumption indicated a comparatively increased role for non-glycolytic oxidative phosphorylation. Up to 38% of equine COC oxygen consumption could be attributed to non-mitochondrial activities and there was a significant loss of spare respiratory capacity over the course of IVM. Notably, our data also revealed that current IVM protocols may be failing to satisfy the metabolic demands of the equine COC. Our findings constitute the first report on mitochondrial efficiency in the equine COC and provide new insight into comparative gamete biology as well as metabolism of the COC during *in vitro* maturation.

## Introduction

Horses serve as valuable models for the study of comparative reproductive biology and are commercially important. However, clinical translation of assisted reproductive technology to the horse remains limited, in large part because conventional *in vitro* fertilisation (IVF) remains unsuccessful^1^. This limitation has in part been circumvented by the application of intracytoplasmic sperm injection (ICSI), which has proven effective for the production of equine embryos *in vitro* and subsequently foals^2,3^ and has consequently gained significant research and commercial interest^4,5^. However, the limitations of assisted reproduction in the horse are compounded because horses are monovular and do not respond well to superovulation protocols^6^. As a result, oocytes for ICSI are typically recovered from immature ovarian follicles and matured in the laboratory (*In vitro* maturation; IVM). *In vitro* maturation is performed in culture media intended to provide physiologically relevant nutritional and physicochemical support to the cumulus-oocyte complex (COC); however, the choice of media used in equine IVM has been informed on the basis of use in other species. Importantly, the specific requirements of equine oocytes during maturation *in vitro* remain largely unknown. Given the clear links between a suboptimal periconceptual environment and negative long-term offspring health outcomes^7^ coupled with the growing application of IVM for clinical application in foal production, it is crucial to gain a better understanding of the intrinsic physiology of the equine COC during IVM.

The most widely used base media are TCM-199, first reported for equine IVM by Zhang *et al*.^8^; and DMEM/F12^3^. The composition of such media differs from the environment *in vitro* and are thus likely to be suboptimal. For example, TCM-199 provides no pyruvate, whereas concentrations in equine follicular fluid range between 0.03–0.13 mM^9^. Pyruvate is important both for oocyte metabolism^10–12^ and for scavenging of reactive O_2_ species (ROS)^13^. The combined presence of glucose and pyruvate results in the highest maturation rates for mouse COCs^14^.

In addition to the lack of knowledge of the optimal culture media to support equine IVM, we know little about the optimal environmental conditions. In this regard, the benefits of a low O_2_ concentration during embryo culture are widely accepted and the use of 5% O_2_ is nearly universal for *in vitro* embryo culture in animals and humans^15^. The optimum O_2_ tension for IVM is still under debate, although in practice, IVM is largely performed in atmospheric oxygen tension^16^. This is despite the fact that the O_2_ concentration in the ovarian follicle is between 1 and 5.5% in the human^17^; the O_2_ tension in the follicle of the horse is unknown and current equine IVM protocols are performed under atmospheric O_2_ (~21%)^3,5,18^.

Identifying optimal conditions for maturation of oocytes *in vitro* is a vital component of successful assisted reproduction. It is well established that *in vitro-*matured oocytes have diminished developmental competency compared to *in vivo* matured^19,20^, an observation that also applies to the horse^18^. The conditions in which any cell is exposed can have a major impact on biochemical and metabolic regulation. In the case of the equine oocyte, a recent study by Walter *et al*.^21^, reported that the protein and metabolite profile in cumulus cells was modified significantly in *in vitro-*matured oocytes compared to those matured *in vivo*. The systems biology approach used by Walter and colleagues highlighted a number of biochemical pathways in cumulus cells that appear to respond to *in vitro* exposure and support the notion that alterations in metabolism may represent a potential mechanism to explain differences in developmental competency^22^. However, the step next required is to explore the dynamic nature of metabolic interaction between the cumulus and the oocyte in the horse under *in vitro* conditions.

In order to satisfy demands for metabolic energy during maturation, the oocyte relies on a finely regulated co-operation with its associated cumulus cells^23,24^. In the mouse, the most widely studied model, the cumulus cells perform glycolysis both for their own ATP production and to provide pyruvate to the oocyte^25^. The oocyte oxidises this pyruvate, in combination with endogenous fuel sources, for ATP production through the TCA cycle and oxidative phosphorylation (OXPHOS) in the mitochondria^26^. Broadly similar profiles exist in the pig^27^ and cow^28^. In contrast, while data are emerging that suggest the pathways used for energy metabolism^21^, the dynamics of metabolism, the preferred substrates for ATP production, or details of mitochondrial function in equine COCs are as yet undefined.

The aim of our studies was to use a novel combination of techniques to measure oxidative mitochondrial metabolism^29^ and consumption/release of energy substrates to enable the comprehensive examination of the glycolytic and oxidative metabolism of equine COCs during IVM. We carried out a systematic examination of cellular and mitochondrial metabolic health during equine IVM and calculated the relative contribution of OXPHOS and glycolysis to overall ATP production. We also examined the metabolic effect of including pyruvate to IVM culture medium, and of reducing the O_2_ concentration during culture, to provide information essential in the optimisation of IVM.

## Results

### Glucose consumption and lactate production

**Figure 1:**
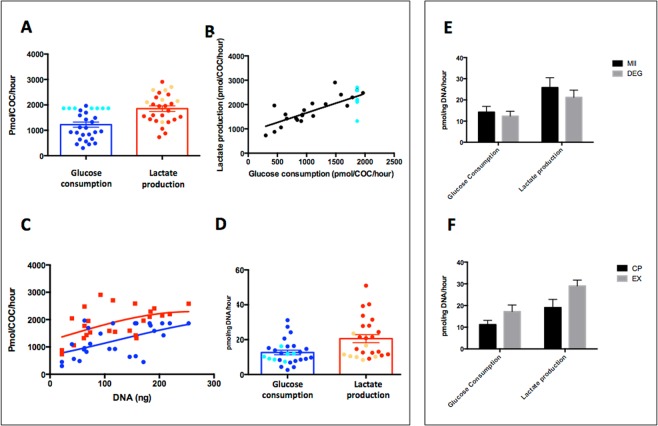
Characterisation of carbohydrate metabolism in equine COCs Data describing glucose consumption (BLUE) and lactate production rates (RED) in equine COCs during *in vitro* maturation (n = 29). Panels (A,D) denote the mean (±SEM) rates for individual COC and are presented per COC/h (**A**) and per ng DNA/h (**D**). Panel (B) shows linear correlation between glucose consumption and lactate production rates (r^2^ = 0.59; p < 0.001). COCs that depleted all available glucose are highlighted separately (aqua [glucose] and orange [lactate]). Panel (C) shows correlation of both glucose consumption and lactate production with DNA content (r^2^ = 0.3; P = 0.01). Glucose consumption and lactate production rates by maturation status (**E**; metaphase II [MII] and degenerated [DEG] oocytes) and cumulus classification (**F**; compact [CP] and expanded [EX]). Bars represent mean ± SEM. (n = 29). There were no statistically significant differences between either maturation status or cumulus classification (p > 0.1).

We first assessed the baseline metabolic activity, in terms of glucose consumption and lactate production, of equine COCs during *in vitro* maturation. Abattoir-derived COCs (n = 29) were cultured undisturbed for 30 h individually in 10-μl droplets of M199-based medium under oil, and the spent medium analysed. The mean overall consumption of glucose was 1223 ± 102.99 pmol/COC/h and the mean overall production of lactate was 1851 ± 109.99 pmol/COC/h (Fig. 1A). On an individual COC basis, the consumption of glucose correlated with the production of lactate (r^2^ = 0.59, p < 0.01; Fig. 1B) and both were correlated to COC DNA content (assessed as a proxy for number of cumulus cells, both r^2^ = 0.3, p < 0.01; Fig. 1C). Therefore, all subsequent analyses were corrected for DNA content. Thus, mean glucose consumption was 12.64 ± 1.3 pmol/ng DNA/h and lactate production were 20.6 ± 2.3 pmol/ng DNA/h (Fig. 1D).

Of the 29 oocytes analysed, 14 progressed to metaphase II (MII, 48%). Equine oocytes that do not mature in culture degenerate, thus oocytes were divided in MII and degenerating at the end of IVM culture. Interestingly, there was no difference in glucose consumption or lactate production between COCs that attained MII compared to those that degenerated during IVM (Fig. 1E). Cumulus density did differ according to morphology; COCs with compact (Cp) vs. expanded (Ex) cumulus differed in DNA content (125 ng/COC vs. 79 ng/COC respectively, p <0.001). However, this difference in morphology did not result in any difference metabolism when normalised for DNA content (p > 0.1; Fig. 1F).

Intriguingly, 7/29 (24%) of COCs depleted all available glucose from the culture medium. In these 7 COCs, only 60% of glucose consumed was accounted for by lactate production, in contrast to 95% for those COCs for which glucose was not limiting (p = 0.02). There was no difference in maturation rate between oocytes that did (4/7, 57.1%) or did not (10/22, 45.4%) deplete all available glucose from the droplet.

### Temporal changes in carbohydrate metabolism and the impact of oocyte presence

**Figure 2:**
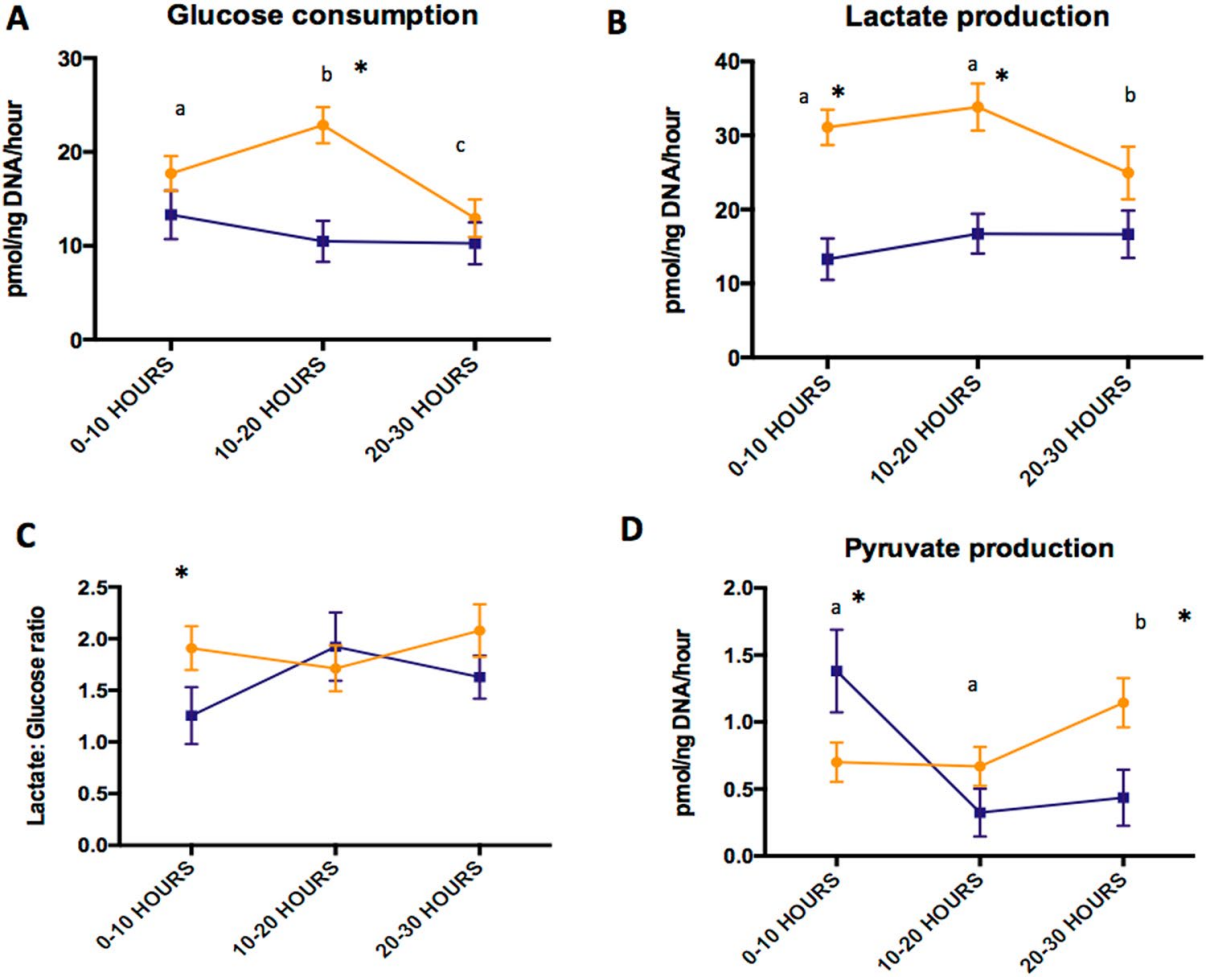
Carbohydrate metabolism of COCs during IVM over time. Effect of oocyte presence (COC; yellow) vs. granulosa cells only (blue) on glucose consumption (**A**), lactate production (**B**), lactate: glucose ratio (**C**) and pyruvate production (**D**) over the time course of IVM. Mean ± SEM are depicted and asterisks represent significant difference between conditions at that time point (n = 56; p < 0.05). Different superscripts indicate differences between time points in the COC data (yellow line**)**.

To assess dynamic temporal changes in COC carbohydrate metabolism during IVM, the concentrations of nutrients (glucose, lactate and pyruvate) in spent medium following the first, second and third 10-h periods of IVM culture were measured. In addition, based on earlier observations that neither glucose consumption or lactate production differed between COCs that contained a mature or degenerated oocyte, the metabolism of intact COCs and isolated granulosa cells were compared at specific timepoints. The patterns and amounts of glucose consumed and lactate produced by COCs was significantly different to those measured from isolated granulosa cells during at least one time period over the 30 h of IVM (n = 56; Fig. 2A–C). Glucose consumption by COCs rose from 0–10 to 10–20 h of culture, with a subsequent decline during hours 20–30. This was accompanied by a concomitant fall in lactate production during the 20–30 h period, the result of which was that the lactate:glucose ratio remained unchanged throughout the period of IVM. In contrast, the consumption of glucose and production of lactate by isolated granulosa cells remained constant over time. We also detected the production of pyruvate by both isolated granulosa cells and intact COCs at various time points during IVM (Fig. 2D). Pyruvate production by COCs was low during hours 0–10 and 10–20 but rose significantly during hours 20–30. Granulosa cells showed the opposite temporal pattern, with pyruvate production decreasing over the time periods. These univariate findings were confirmed in a mixed multivariable linear regression model (SI Tables 1 and 2).

### Basal O_2_ consumption rate and mitochondrial function

**Figure 3:**
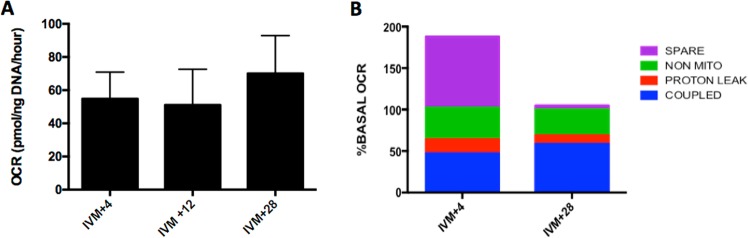
Oxidative metabolism of COCs during IVM over time. Mean (± SEM) Basal O_2_ consumption rates (OCR) measured at 4, 12 and 28 h after the initiation of the 30 h *in vitro* maturation period (**A**; IVM +4, +12, +28) (n = 12 COCs). Mitochondrial function at IVM + 4 h (outset) and IVM + 28 h (end) as determined from respiratory inhibitor studies (**B**; n = 57 COCs). OCR coupled to ATP production (BLUE), OCR associated with proton leak (RED), non-mitochondrial OCR (GREEN) and spare respiratory capacity (PURPLE) are expressed as % basal OCR. Mean values are stacked. There was a significant difference between spare capacity at time IVM +4 and IVM +28 h; P<0.01.

After identifying the consumption and release of key metabolic substrates, the oxygen consumption rate (OCR), which is a measure mitochondrial function, of COCs throughout IVM was determined. Given the findings that consumption/release of substrates varies throughout IVM, representative time points of 4 h, 12 h, and 28 h were chosen to reflect the 0–10 h, 10–20 h and 20–30 h windows. Basal OCR (mean 58.6 ±10.9 pmol/ng DNA/h) remained constant over time (n = 12; Fig. 3A). At 4 and 28 h, mitochondrial function was assessed by the sequential addition of respiratory inhibitors (n = 69). OCR coupled to ATP production (47 ± 10% and 58 ± 4% at 4 and 28 h respectively), non-mitochondrial OCR (38 ± 10% and 31 ± 5% at 4 and 28 h respectively) and OCR attributed to proton leak (17 ± 6% and 17 ± 2%) did not vary between 4 and 28 h (Fig. 3B). However, respiratory spare capacity was notably higher at 4 h (85 ± 31%) than at 28 h (5 ± 3%; p < 0.01; Fig. 3B).

### Effect of pyruvate addition and O_2_ concentration on glucose metabolism, basal O_2_ consumption rate and mitochondrial function

**Figure 4:**
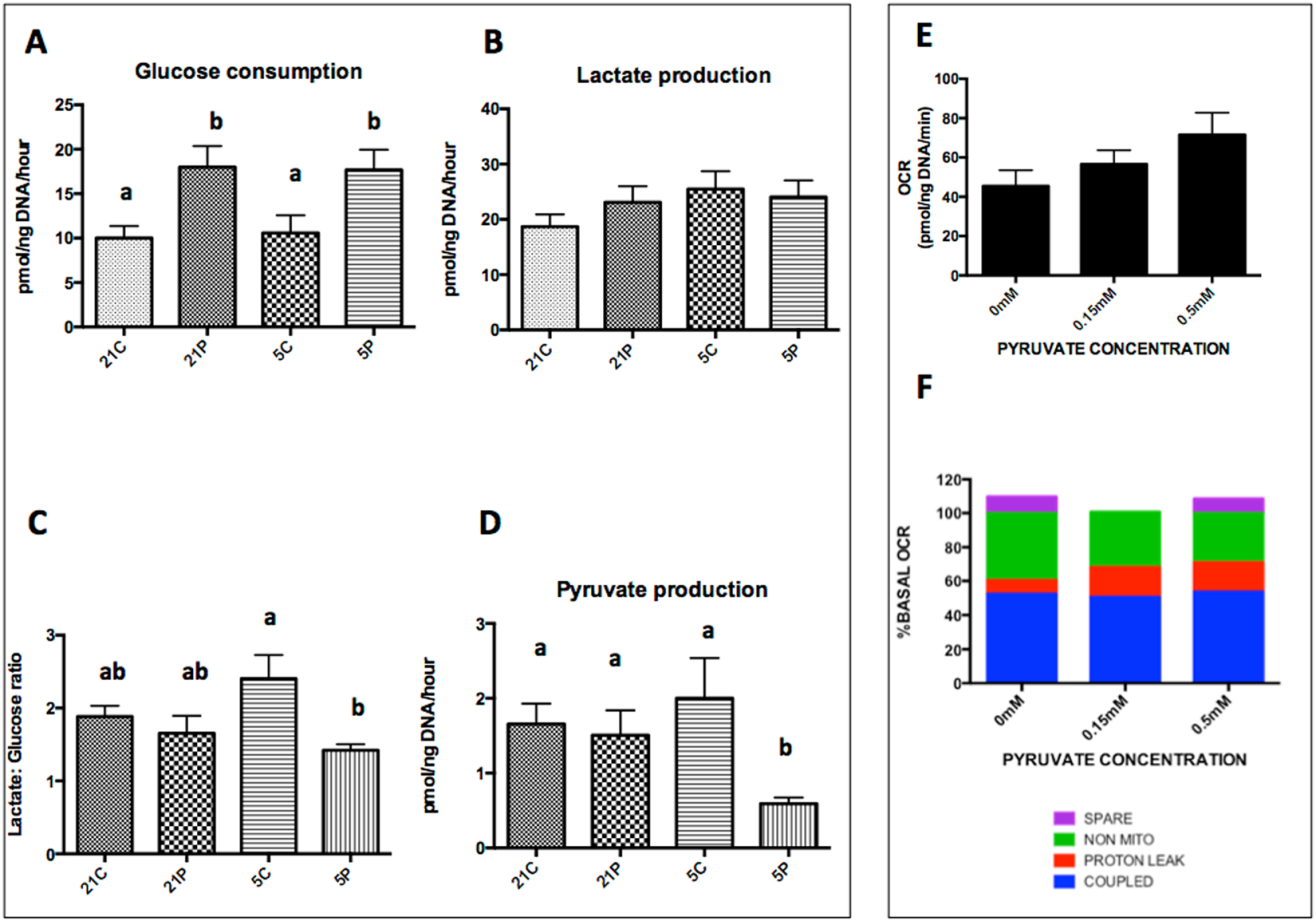
The effect of 0.15 mM pyruvate and O_2_ concentration on carbohydrate and oxidative metabolism of COCs during IVM. The effect of 0.15 mM pyruvate and O_2_ concentration (5% vs. 21%) on glucose consumption (**A**), lactate production (**B**), lactate:glucose ratio (**C**) and pyruvate production (**D**) during IVM. Condition abbreviations on X axes are as follows; 21C = Control maturation medium and maturation at 5% CO_2_ in air (21% O_2_); 21 P = Pyruvate containing Maturation Medium (control medium as above with the addition of 0.15 mM Pyruvate) and incubation at 5% Co_2_ in air (21% O_2_); 5C = Control maturation medium and maturation at 5% CO_2_, 5% O_2_ and 90% N_2_; 5 P = Pyruvate containing maturation medium and incubation at 5% CO_2_, 5% O_2_ and 90% N_2_. Bars represent mean ± SEM and different superscripts denote significance differences between groups (P < 0.05**)**. Effect of medium pyruvate concentration (0, 0.15, 0.5 mM) during IVM on basal OCR (**E**; n = 45). Mean ±SEM are presented. Effect of pyruvate concentration during IVM on mitochondrial function as determined following the sequential addition of respiratory inhibitors (**F**; n = 45). O_2_ consumption rate (OCR) coupled to ATP production (BLUE), OCR associated with proton leak (RED), non-mitochondrial OCR (GREEN) and spare respiratory capacity (PURPLE) are expressed as % basal OCR. Mean values are stacked and there were no significant differences between groups.

Having described the profile of consumption and release of energy substrates in tandem with mitochondrial activity throughout IVM in standard culture conditions, we next examined the extent to which metabolism responds to a modified culture environment. COCs (n = 132) were cultured in standard equine IVM conditions (detailed in methods and referred to as control medium) or in medium supplemented with pyruvate (0.15 mM). In addition, we examined the interaction with culture under high (21%) and low (5%) ambient O_2_ (Fig. 4). Cumulus was trimmed, and droplet size increased to 15 μl, in an attempt to prevent glucose from becoming limiting. The proportions of oocytes reaching MII was unaffected by the presence of pyruvate or O_2_ concentration (35–43.5%; p> 0.05). In each experimental condition, there were COCs that depleted all available glucose, despite trimming the cumulus and increasing the droplet size. Interestingly, in the control group (high O_2_ without pyruvate) only 1/35 (2.9%) COCs depleted all glucose; a significantly lower proportion (p<0.05) than in the other three experimental groups (16.2% high O_2_ with pyruvate, 22.2%, low O_2_ with pyruvate and 26.1% in low O_2_ without pyruvate).

The effect of O_2_ concentration and supplementation with pyruvate on glucose consumption and lactate production was evaluated in COCs for which glucose was not limiting (n = 103). Independent of O_2_ tension, in presence of 0.15 mM pyruvate, COCs consumed significantly more glucose, without a corresponding increase in lactate production (Fig. 4A,B), this resulted in a decreased lactate:glucose ratio in the low O_2_ with pyruvate group (Fig. 4C). Even when in pyruvate-replete conditions, equine COCs released pyruvate into the spent medium (Fig. 4D). Moreover, there was no association between pyruvate production and COC DNA content. Those COCs cultured in low O_2_ in pyruvate-replete culture medium produced less pyruvate than COCs in each of the three other conditions (0.6 vs. 1.6–2.1 pmol/ng DNA/h; Fig. 4D, p = 0.02). These univariate findings were confirmed using multivariable linear regression modelling (SI Table 3).

Interestingly, the presence of 0.15- or 0.5-mM pyruvate during IVM in 21% oxygen (n = 45 COCs) had no significant effect on basal OCR (Fig. 4E). Moreover, the components of OCR were unaffected by the addition of pyruvate (Fig. 4F).

## Discussion

We report the most detailed comprehensive characterisation of glucose, lactate and pyruvate metabolism, O_2_ consumption and mitochondrial efficiency in equine COCs to date. The data reveal that the metabolism of the equine COC is unique compared to the COCs from other mammals. Aerobic glycolysis is the major pathway of glucose metabolism in equine COCs during IVM, with 95% of glucose consumed able to be accounted for by lactate production in COCs. Intriguingly, our data also revealed a sub-population of equine oocytes that depleted all glucose provided in the culture medium. While the bulk of glucose consumed could be accounted for by lactate production, the high concurrent OCR demonstrates that OXPHOS plays a significant role in energy metabolism in equine COCs. The identity of substrates for OXPHOS are likely to include a portion of the glucose not accounted for in terms of lactate production, as well as amino acids and fatty acids although further investigation is required to confirm this. Importantly, we also observed a progressive decline in mitochondrial function as IVM progressed. Having established a profile of glycolytic and oxidative metabolism in equine oocytes during IVM, we further explored the extent to which this can be modified in response to changing the IVM culture conditions. The addition of pyruvate to the IVM medium led to a significant rise in glucose consumption, without a concomitant lactate response, indicating that equine COC metabolism is highly sensitive to the composition of the culture medium.

In the single previous report on glucose metabolism by equine COCs^30^, the state of cumulus expansion appeared to influence glucose consumption; oocytes that had a fully expanded cumulus were found to have markedly lower glucose consumption during IVM. However, the metabolic data was not normalised for cumulus cell number, which we found to be significantly lower in expanded COCs, and which may help to explain the findings reported by Gonzalez-Fernandez *et al*. As reported by Sutton *et al*.^31^ in cattle, normalising metabolic data for DNA content (i.e., per cumulus cell) allows for more accurate comparison of metabolic measurements among COCs, given the wide range of COC mass related to the number of cells and their state of expansion. When adjusted for DNA content, we observed that the mean glucose consumption and lactate release during IVM by equine COCs were approximately half of those values reported in bovine COCs^28^. In light of this, it was interesting that the total OCR of equine oocytes in our study was ~20 times higher than that reported for bovine COCs^28^. Our data are supported by the high reported resting total OCR (180 pmol/oocyte/hour) for equine denuded oocytes^32^; a value which is 6–10 times that for denuded bovine, human and porcine oocytes^27,33,34^. Furthermore, we have previously reported that equine total COC OCR was 10 times higher than bovine COC OCR in the same conditions^29^. Combined, these data reflect a significant and marked species difference in COC metabolism between equine and other mammalian COCs studied thus far, and indicate that OXPHOS makes a major metabolic contribution in the female equine gamete. It is noteworthy that stallion sperm metabolism also differs from that of other species, with an apparent reliance OXPHOS and not glycolysis for both motility and membrane integrity^35^.

Measuring consumption and release of substrates alongside OCR allows an estimate to be made of overall ATP production by equine COCs. The production of one molecule of lactate equates to the net production of one molecule ATP, whereas the consumption of one mole of O_2_ produces approximately five moles of ATP^36^. On this basis, the equine COC produces approximately 87% of ATP from OXPHOS with 13% from glycolytic production of lactate. This pattern contrasts with the bovine COC, which, under similar conditions produces only around 20% of ATP from OXPHOS^28^. Considering substrate utilisation, the glycolytic index was typically close to 100%, suggesting that the major metabolic fate of glucose was the release of lactate and that other substrate(s) are oxidised to generate ATP in equine COC undergoing IVM. Candidate substrates include lipid, amino acids and glycogen. While intracellular lipid content has not been directly measured in equine oocytes, they show poor cryotolerance and have notably dark cytoplasmic inclusions, as in the pig, which ultra-structurally appear as abundant lipid-like vesicles^37^, indicative of a significant ooplasmic lipid reserve. Taken together, these observations suggest that fatty acid oxidation (FAO) may play a major role in ATP generation in the equine oocyte. This presents a fascinating avenue for further study.

While basal OCR remained stable throughout IVM, glucose metabolism did appear to fluctuate, with equine COCs consuming glucose and producing lactate at a lower rate during the final 10 h of IVM. However, the lactate:glucose ratio remained constant, suggesting an overall decrease in glucose metabolism rather than a change in metabolic pathways. Studies in other species have produced conflicting results on temporal changes in metabolism over the course of IVM^28,38–40^.

Germinal vesicle breakdown (GVBD), a key component of meiosis, has been associated with an increase in glucose consumption via glycolysis and the pentose phosphate pathway in mice^41^. We observed a significant rise in glucose consumption during hours 10–20 of IVM, which coincides with the timing of GVBD in horse^42,43^. Notably, when granulosa cells were cultured alone there was no variation in glucose and lactate metabolism in hours 10–20  of culture, suggesting that the oocyte and/or associated corona radiata cells influence energy requirements in the COCs, supporting the notion that it is oocyte-derived activities that drive the increase in glucose consumption during IVM. In stark contrast to this observation, the metabolism of COCs containing degenerated oocytes did not differ from those containing a MII oocyte.

The determination of mitochondrial function by OCR has been reported widely for somatic cells, but there are fewer reports in oocytes. Of the studies that have been performed, the majority have been conducted on denuded oocytes (bovine^29,33^, equine^32^). To our knowledge, there is only one prior report on intact COCs, using both bovine and equine^29^. However the current study reveals, for the first time, the specific components of mitochondrial function in intact equine COCs. Our data indicated that approximately half of the oxygen consumed by intact equine COCs was coupled to synthesis of ATP; a finding much lower to the 85% reported in equine denuded oocytes^32^, but similar to that of bovine COCs (43% ± 4.1;^29^). Coupled oxygen consumption did not change over the time course of IVM; a pattern similar to that reported in intact bovine COCs^29^ but again in contrast to the bovine denuded oocyte, in which coupling efficiency decreased from 40.9% in immature oocytes to 25% in MII oocytes^33^. The fall in coupling efficiency in denuded bovine oocytes was attributed to an increase in proton leak, a term that describes a passage of protons across the mitochondrial membrane without synthesising ATP, and is an important regulator of ROS. Fascinatingly, we observed that the proportion of O_2_ that can be accounted for by proton leak in the equine COC was low and did not vary during IVM. A further important finding in our studies was that not all O_2_ was used to support ATP synthase activity; our data revealed that 38% of equine COC O_2_ consumption could be attributed to non-mitochondrial activities. Non-mitochondrial oxygen consumption may indicate activity of O_2_-consuming cytosolic enzymes such as NADPH oxidase, xanthine oxidase and squalene mono-oxygenase, as demonstrated in rabbit embryos^44^. By contrast, somatic cells typically have a non-mitochondrial OCR of approximately 10% of basal^45^.

Spare respiratory capacity represents the ‘scope’ by which a cell can increase its oxidative metabolism, and by implication, the smaller the spare capacity, the closer a cell is to working at its bioenergetic limit. In the present study we discovered an 80% reduction in the spare respiratory capacity of equine COCs at the end of IVM, however the observed increase in OCR could not account for all of this loss of spare capacity. A decrease in spare capacity is a marker of mitochondrial dysfunction^46^ which potentially, has detrimental consequences for oocyte developmental competence. In our earlier work, intact bovine COCs maintained a consistent spare capacity^29^, while Sugimura *et al*.^33^ reported that bovine denuded aged oocytes exhibited a decreased spare capacity following IVM. In the horse however, oocytes *in vivo* are viable for 6–12 h after ovulation indicating that oocyte aging is unlikely to be responsible for the reduction in spare capacity observed herein after 28 hours of IVM^47,48^. However, it is possible that the cumulus cells, nearing the end of their physiological life have no further requirement to maintain ‘metabolic scope’ and that this loss of mitochondrial spare capacity signals their demise^49^. To fully understand the relevance of the loss of spare capacity, comparisons should be made with contemporaneous *in vivo* COCs, however, our data do indicate that current IVM culture may be having deleterious effects on the metabolic regulation of the equine COCs.

One unexpected finding from our study was that around 1 in 4 COCs depleted all the available glucose from a 10-μl droplet of maturation media during IVM. This was surprising, since the medium used for equine IVM is based on TCM199, and as such contains 5.6 mM glucose. This exhaustion of glucose has not been previously reported, but does indicate that this commonly used IVM system, i.e. M199 at a ratio of 10 µL medium per COC^50–52^ may be inadequate in terms of glucose supply. It was noteworthy that in examining the effect of pyruvate and O_2_ concentration, the granulosa was tightly trimmed and the volume of the droplet increased to 15 μl which resulted in only 3% (1/35) of COCs in the control group exhausting all available glucose. This suggests that while the number of cumulus cells are partly responsible for the exhaustion of glucose as shown in Fig. 1C, there is still metabolic adaption occurring in those COCs as demonstrated by the reduced glycolytic index. A recent report^21^ which examined the *cumulome* of equine *in vivo* vs. *in vitro* -matured oocytes found that glycolytic metabolites and proteins were higher in the *in vitro* samples, suggesting some degree of adaptation to the *in vitro* environment. These authors also reported greater amounts of NADH-ubiquinone oxidoreductase chain 5 protein (mt ND5) in the cumulus of IVM oocytes. This protein is a key component of the respiratory chain, suggesting an increased reliance on OXPHOS. Importantly, our data offers the first insight into the physiological function of equine COCs in an *in vitro* setting, and our observations of metabolic adaptation to the sub-optimal *in vitro* environment are reinforced by the data reported by Walter *et al*. While maturation rate was not seemingly affected by these adaptations, further studies following these oocytes through fertilisation and embryo culture are required to establish if developmental competence is affected by this metabolic adaptation. It would also be interesting to evaluate ROS and lipid content in the COCs that exhausted all glucose to ascertain which alternative pathways were activated.

Our data have indicated that equine COCs adapt their biochemical process in response to maturation *in vitro*, and that current, widely used conditions for IVM may be failing to satisfy the needs of the equine COC. To attempt to improve IVM, we modified the medium through the inclusion of pyruvate, and reduced the ambient O_2_ concentration. Pyruvate was added due to its multiple roles; both as a confirmed energy substrate for COCs^53^ and since it can act as an antioxidant, via its α-keto carboxylate structure which to directly neutralizes ROS^54,55^. Antioxidants have been shown to benefit both embryo and oocyte culture^56^. We simultaneously reduced the ambient oxygen concentration to 5%. High O_2_ is confirmed to be deleterious for embryo development^15^, but the jury is out on its effect on oocytes which are traditionally cultured these in 21% O_2_ rather than 5%^16,57,58^. Addition of pyruvate (0.15 mM) led to an increased glucose consumption without affecting maturation rate, O_2_ consumption or mitochondrial function. This finding was unexpected since pyruvate is a product of glycolysis, where it can be converted into lactate and excreted from the cell, or converted to acetyl Co A to take part in TCA cycle. It was expected that the provision of pyruvate in the *in vitro* maturation medium would spare glucose, due to the predicted ease of transport of pyruvate. Leese and Barton (1985)^53^ established that mouse oocytes consume pyruvate; however, equine COCs produced pyruvate in the present IVM system the source of which is presumably glucose as there was minimal lactate in the starting medium (<1 mM from FBS). These findings suggest that the supplementation of IVM medium with pyruvate prompted an increase in glycolysis, without affecting OXPHOS rates and lead us to conclude that current IVM systems are inadequate for the metabolic regulation of equine oocytes.

In conclusion, we have reported the most detailed examination of equine COC metabolism *in vitro* to date. The metabolic profile of the female equine gamete differs markedly from the patterns observed in the oocytes of other mammals studied. Because of our findings, we conclude that current approaches for *in vitro* maturation of equine gametes fail to satisfy the unique metabolic needs of the oocyte, and as such there is an urgent need for a concerted effort to design better culture medium to better meet demands. Whilst current protocols have been successful in terms of the ability to generate live foals, there has been a reliance on ICSI, and the failure of IVF may also point to inadequate maturation protocols. We highlight the value of these advanced metabolic studies in understanding not only the basic physiology of the COC, but the effect of clinical manipulation on that physiology which can be extrapolated to any species, particularly given the rise in clinical interest in human IVM.

## Methods

### General methods

Ovaries were harvested within 15 minutes of slaughter from mares of unrecorded age and breed. Animals were slaughtered during the natural breeding season (April-October in the UK) in compliance with EU legislations EC 852/2004, 853/2004 and 854/2004, for purposes unrelated to the study. Cumulus-oocyte complexes were recovered as previously described^59^ and classified on the basis of cumulus morphology as denuded or corona only, compact (Cp) or expanded (Ex). Only Ex and Cp COCs were used. Recovered COCs were placed in groups of 10 in 1-ml glass vials in EH Medium (40% M199 with Hanks salts, 40% M199 with Earle’s salts [Life technologies Ltd.], 20% foetal bovine serum (FBS) and 25 μg/ml gentamicin^60^. Vials were held overnight (12–18 h) at 20–24 °C. All maturation dishes, pre-prepared with 9-μl droplets of experimental media covered with an oil overlay were pre-equilibrated in experimental conditions at 38.3 °C for 12 h before use.

### Glucose consumption and lactate production during IVM

Maturation was carried out in individual 10-μl droplets of Maturation Medium (M199 with Earle’s salts, 10% FBS, 25 μg/ml gentamicin with 5 mU/ml FSH [Sioux Biochemical Inc., Sioux centre, IA] under oil, with nine droplets per dish. Three medium-only droplets in each dish were used as reference droplets. After the 30-h maturation period, oocytes were denuded of cumulus in 80 IU/ml hyaluronidase, and classified as MII (polar body present), intact (intact oolemma without a polar body) or degenerated (DEG). Denuded cumulus cells from each COC were placed in wells of a 96-well plate and kept at −20 °C for DNA quantification of cumulus cells^28^. Spent media was frozen at −80 °C and analyses of thawed samples was carried out using enzyme-linked assays and the consumption and release method^61^.

### Temporal changes in GPL metabolism during IVM and the impact of oocyte presence

COCs were individually cultured and dishes prepared as described above, however each individual COC was moved to a fresh droplet at 10-h intervals. In each replicate, half of the droplets contained mural granulosa cells only, estimated to be the same volume as the COC. Mural granulosa cells were obtained from dishes in which a Cp COC was located (i.e. were from the same follicle). The oocytes were classified, and cells and spent media were analysed, as described above. DNA content was quantified from granulosa and denuded cumulus cells.

### Basal O_2_ consumption rate and mitochondrial function during IVM

OCR was determined using an Agilent Seahorse XFp analyser^29^. Groups of three Cp COCs were cultured for IVM in Seahorse plates in 180 μl of maturation medium. Mitochondrial function was evaluated at either 4 or 28 h. To do this, after calibration, three basal O_2_ consumption rate readings (pmolO_2_/well/min), were recorded before a series of respiratory inhibitors were sequentially added to each well; 1) Oligomycin [1 μM] 2) FCCP [5 μM] 3) Antimycin/Rotenone [2.5 μM]. After the readings were obtained, DNA content was quantified from COCs.

### Effect of pyruvate addition and O_2_ concentration during IVM on GPL metabolism

Maturation (containing equal numbers of Cp and Ex) was carried out in individual 15-μl droplets in control Maturation Medium or with 0.15 mM added Pyruvate, in either 5% CO_2_ in air (21% O_2_) or in 5% CO_2_, 5% O_2_ and 90% N_2_ (5% O_2_). After maturation, COCs and media were processed as described.

### Effect of pyruvate addition during IVM on oxidative metabolism

*In vitro* maturation was carried out directly in a Seahorse plate in Maturation Medium with 0, 0.15 mM or 0.5 mM added Pyruvate, before basal OCR and mitochondrial function testing was performed. After the readings were obtained, COCs were processed as described.

### Statistical analyses

#### Carbohydrate metabolism

The primary outcome variables were glucose consumption, lactate production, and pyruvate production (expressed as pmol/ng DNA/hour). Glycolytic index was calculated as the ratio of lactate production/glucose consumption (a value of 2 is 100%). Significance was set at p <0.05 and all data are presented as mean ±SEM. One-way ANOVA was used to explore differences between time points or conditions, with Student’s test used when only two groups were compared. Mixed multivariable logistic regression models were used with COC ID included as a random effect to account for repeated measures over time when evaluating temporal changes in glucose metabolism and when evaluating the effect Pyruvate and O_2_ multivariable linear regression models were used. In performing the analysis, all potential explanatory variables were included initially, and subsequently retained if they improved model fit. Variables were selected for inclusion if the p<0.25 threshold was attained after a backward-stepwise method. We used the likelihood ratio test to compare all competing models.

#### O_2_ consumption rate measurement

The third reading was used for all basal OCR readings and the reading representing maximal response was used for all inhibitor OCR readings. Data were expressed as pmol O_2_/ COC/ h or pmol O_2_/ng DNA/h. Percentage data were first arcsine transformed before performing either one-way ANOVA or Student’s test to explore differences between time points or conditions.

## Supplementary information

Supplementary Information.

## Data Availability

The authors confirm that all source data will be deposited on an institutional data repository and made available upon request to the senior author.
