# Supplementary material for: Energy metabolism of the equine cumulus oocyte complex during in vitro maturation
Source: Sci Rep. 2020 Feb 26;10:3493. doi: 10.1038/s41598-020-60624-z (PMC7044441; doi:10.1038/s41598-020-60624-z)
Supplement: Supplementary file 1 — Supplementary Information. [file 41598_2020_60624_MOESM1_ESM.pdf]

Energy metabolism of the equine cumulus oocyte complex during *in vitro* maturation

Niamh Lewis*, Katrin Hinrichs, Henry J Leese, Caroline McGregor Argo, Daniel R Brison, Roger Sturmey

Supplementary Table 1: Temporal changes in glucose metabolism over the course of IVM

	Variable	Sub Category	Coef.	95% CI		P
**Glucose Consumption**	**Time**	**0-10h**	Ref.			
		**10-20 h**	4.31	0.95	7.66	0.01
		**20-30 h**	-5.17	-8.49	-1.85	0.002
	**Batch**	**1**	Ref.			
		**2**	-6.8	-12.48	-1.13	0.02
		**3**	5.09	0.69	9.5	0.02
		**4**	-6.43	-12.12	-2.75	0.002
		**5**	-5.81	-10.76	-0.85	0.02
		**6**	2.78	-1.9	7.47	0.24
	**Constant**		17.41	13.49	21.32	<0.001
**Lactate production**	**Time**	**0-10h**	Ref.			
		**10-20 h**	3.14	-0.61	6.88	0.1
		**20-30 h**	-8.28	-12.07	-4.48	<0.001
	**Batch**	**1**	Ref.			
		**2**	-2.67	-14.29	8.95	0.65
		**3**	-13.05	-22.08	-4.02	0.01
		**4**	-10.52	-20.16	-0.9	0.03
		**5**	2.89	-7.22	13.02	0.58
		**6**	-2.93	-12.51	6.63	0.55
	**Constant**		36.56	29.28	43.84	<0.001
**Pyruvate production**	**Time**	**0-10h**	Ref.			
		**10-20 h**	0.73	-0.44	0.59	0.78
		**20-30 h**	1.05	0.52	1.58	<0.001
	**Batch**	**2**	Ref.			
		**3**	-0.88	-1.81	0.05	0.06
		**4**	-0.32	-1.23	0.6	0.5
		**5**	0.21	-0.73	1.14	0.67
		**6**	0.35	-0.55	1.25	0.45
	**Constant**		1.52	0.81	2.23	<0.001

Table 1 continued

	Variable	Sub Category	Coef.	95% CI		P
**Lactate: Glucose ratio**	**Time**	**0-10h**	Ref			
		**10-20 h**	-0.21	-0.41	0.37	0.92
		**20-30 h**	0.29	-0.11	0.69	0.16
	**Batch**	**1**	Ref			
		**2**	1.17	0.38	1.96	0.004
		**3**	-0.98	-1.51	-0.46	<0.001
		**4**	0.84	0.26	1.42	0.004
		**5**	1.33	0.74	1.93	<0.001
		**6**	-0.3	-0.86	0.26	0.3
	**Constant**		1.93	1.45	2.41	<0.001

Supplementary table 1: : Mixed random effect multivariable linear regression model for effect of time during IVM on glucose consumption, lactate and pyruvate production and lactate : glucose ratio. COC ID was added as a random effect to account for repeated measures over time. Constant represents COC in batch 2 during 0-10 hours of *in vitro* maturation.

Supplementary Table 2: The impact of oocyte presence on glucose metabolism (intact COC vs. granulosa only).

	Variable	Sub-Category	Coef.	95% CI		P
**Glucose consumption**	**Time**	**0-10h**	Ref			
		**10-20 h**	-2.83	-7.93	2.27	0.28
		**20-30 h**	-3.04	-8.14	2.06	0.24
	**Intact COC**		2.34	-3.35	8.03	0.42
	**Time*COC**	**0-10 h*COC**	Ref			
		**10-20h*COC**	10.24	3.65	16.83	0.002
		**20-30h*COC**	-1.31	-7.86	5.25	0.7
	**Constant**		13.31	8.89	17.73	<0.001
**Lactate production**	**Time**	**0-10h**	Ref			
		**10-20 h**	3.91	-1.61	9.41	0.17
		**20-30 h**	3.82	-1.69	9.34	0.17
	**Intact COC**		17.61	9.64	25.58	<0.001
	**Time*COC**	**0-10 h*COC**	Ref			
		**10-20h*COC**	1.01	-6.04	8.07	0.78
		**20-30h*COC**	-9.18	-16.23	-2.12	0.01
	**Constant**		12.81	6.6	19.04	<0.001
**Pyruvate production**	**Time**	**0-10h**	Ref			
		**10-20 h**	-1.89	-2.85	-0.94	<0.001
		**20-30 h**	-1.78	-2.72	-0.84	<0.001
	**Intact COC**		-1.3	-2.36	-0.24	0.02
	**Time*COC**	**0-10 h*COC**	Ref			
		**10-20h*COC**	1.84	0.58	3.1	0.003
		**20-30h*COC**	2.75	1.49	4	<0.001
	**Constant**		2.66	1.87	3.45	<0.001
**Lactate:glucose ratio**	**Time**	**0-10h**	Ref			
		**10-20 h**	0.74	0.18	1.31	0.01
		**20-30 h**	0.34	-0.22	0.9	0.23
	**Intact COC**		0.72	0.31	1.42	0.04
	**Time*COC**	**0-10 h*COC**	Ref			
		**10-20h*COC**	-0.95	-1.68	-0.23	0.01
		**20-30h*COC**	0.06	-0.68	0.79	0.88
	**Constant**		1.22	0.7	1.75	<0.001

Supplementary Table 2: Mixed multivariable random effects linear regression model for impact of oocyte presence (intact COC vs. granulosa only) on glucose consumption, lactate and pyruvate production and lactate : glucose ratio. * Indicates interaction term. Constant represents granulosa only during 0-10 hours of *in vitro* maturation.

Supplementary Table 3: Effect of oxygen concentration and pyruvate concentration during IVM on glucose metabolism.

		Coef.	95% CI		P
**Glucose consumption**	**Media**	7.17	0.53	13.82	0.04
	**Oxygen**	-1.1	-7.22	5	0.72
	**Media*oxygen**	0.78	-7.56	9.13	0.85
	**Trimming**	5.19	1.16	9.23	0.01
	**Constant**	3.07	-4.92	11.06	0.45
**Lactate production**	**Media**	-1.7	-11.13	7.69	0.72
	**Oxygen**	-7.07	-15.71	1.56	0.12
	**Media*oxygen**	6.4	-5.46	18.26	0.29
	**Constant**	25.76	18.67	32.84	<0.001
**Pyruvate production**	**Media**	-1.47	-2.47	-0.48	0.01
	**Oxygen**	-0.52	-1.43	0.4	0.27
	**Media*oxygen**	1.15	-0.1	2.39	0.07
	**Cumulus classification**	0.49	-0.12	1.1	0.11
	**Trimming**	1.22	0.62	1.82	<0.001
	**Constant**	0.09	-1.11	1.29	0.88
**Lactate: Glucose ratio**	**Media**	-0.67	-1.19	-0.16	0.01
	**Oxygen**	-0.2	-0.69	0.3	0.43
	**Media*oxygen**	0.37	-0.28	1.02	0.26
	**Trimming**	-0.47	-0.78	-0.16	<0.001
	**Constant**	2.77	2.17	3.37	<0.001

Supplementary Table 3: Multivariable linear regression model for effect of media (0 mM pyruvate vs. 0.15 mM Pyruvate) and oxygen concentration (5% vs. 21%) during IVM on glucose consumption, lactate and pyruvate production and lactate : glucose ratio. * Indicates interaction term. Constant represents a visually trimmed compact COC matured in control maturation media (0 mM pyruvate) at 21% Oxygen.
